# What’s in a name?

**DOI:** 10.1038/sdata.2018.92

**Published:** 2018-05-22

Shakespeare’s Juliet famously did not consider names as being of any importance. Had she ever needed to track data back to their source, however, she may have felt very differently.

When a scholarly publication refers to a dataset hosted at a public repository, it is best practice to acknowledge the hosting repository by including its name and other key information in a formal data citation (https://go.nature.com/2qBoCU6). During our standard editorial checks of data citations prior to publication, we routinely encounter inconsistencies in repository naming. We strive to fix these before publication, but the official name of a data repository is not always as clear as might be expected. Naming inconsistencies also pose a challenge as we expand and maintain our list of recommended data repositories (http://go.nature.com/2eLHBFP).

Today, we are calling on the repositories with whom we work to check that their repository’s name is consistently recorded on their webpages, and in any indices that track their repository or datasets. Repository names should be consistently reflected on individual data records, along with other citation information, in both human readable and machine accessible formats. A roadmap describing how repositories can best support data citation is currently open for public comment^1^. For repositories that assign DataCite DOIs to their archived data^2^, the repository name should be recorded in the ‘publisher’ field of each DOI they register, in exactly the form that it should be included in formal data citations.

Repository names should also be consistently recorded in any lists, indexing services or databases that track the repository or its contents. Repositories included on our recommended list should check that we have listed the name they want to be included in data citations at the journal, and that this matches exactly the name they have registered with DataCite, if relevant.

We encourage repository managers to create and maintain a record for their resource at FAIRsharing (https://fairsharing.org/)^3^ and re3data (https://re3data.org/)^4^, two repository indexing services that provide central locations for researchers to search for relevant repositories, and access key metadata about the resource. The curators working at FAIRsharing additionally index standards and data sharing policies, and interconnect these with relevant data repositories. Several publishers and journals maintain collections of the standards and repositories they endorse at FAIRsharing (view ours at https://fairsharing.org/recommendation/ScientificData). These endorsements can be viewed on the individual standard and repository record pages, and can serve as a rough measure – independent of literature citations – of how widely adopted or used a particular standard or resource may be.

Scientific Data authors (and ideally anyone formally citing data), should ensure that they use the repository name that has been registered in the DataCite metadata for the DOI they are citing. If the dataset does not have a DOI, authors may refer to FAIRsharing or re3data for the repository name. Authors should contact the host repository if they encounter any inconsistencies between these records.

Ensuring consistency in data citations will help to increase the stability of research data infrastructure, leading to more reliable data for all stakeholders. We think even Juliet would have appreciated the importance of repository name consistency between repositories, publications, and repository registries for data citations!

(Not up on your Shakespeare? see ref. 5).
